# Heterogeneity in Stage IV Ovarian Cancer: Survival Trends in Patients Presenting with Sister Mary Joseph’s Nodule

**DOI:** 10.3390/cancers18060897

**Published:** 2026-03-10

**Authors:** Ori Tal, Andreas Schötzau, Nicolò Bizzarri, Giacomo Guidi, Anna Fagotti, Intidhar Labidi-Galy, Tally Levy, Viola Heinzelmann-Schwarz

**Affiliations:** 1 Department of Gynecological Oncology and Gyn. Cancer Center, University Hospital Basel, 4031 Basel, Switzerland; info@eudox.ch (A.S.); viola.heinzelmann@usb.ch (V.H.-S.); 2 Gray Faculty of Medicine, Tel Aviv University, Tel Aviv 69978, Israel; 3 UOC Ginecologia Oncologica, Dipartimento per la Salute della Donna e Del Bambino e della Sanità Pubblica, Fondazione Policlinico Universitario a. Gemelli, IRCCS, 00168 Rome, Italy; nicolo.bizzarri@policlinicogemelli.it (N.B.); giacomo.guidi@guest.policlinicogemelli.it (G.G.); anna.fagotti@policlinicogemelli.it (A.F.); 4 Department of Oncology, Hopitaux Universitaires De Geneve, 1205 Geneva, Switzerland; intidhar.labidi-galy@hcuge.ch; 5 Division of Gynecologic Oncology, Edith Wolfson Medical Center, Holon 5822012, Israel; levyt@wmc.gov.il

**Keywords:** ovarian neoplasms, umbilicus, neoplasm metastasis, peritoneal neoplasms, prognosis

## Abstract

**Background/Objectives**: Sister Mary Joseph’s nodule (SMJN) is classified as FIGO stage IVb in ovarian cancer. While traditionally considered an indicator of poor prognosis, emerging evidence suggests variable outcomes. This study aimed to describe overall survival (OS) and recurrence-free survival (RFS) in ovarian cancer patients presenting with SMJN and to explore outcome patterns relative to other FIGO IVb presentations. **Methods:** We conducted a retrospective multicenter cohort study including patients with epithelial ovarian, fallopian tube, or primary peritoneal carcinoma diagnosed between 2010 and 2023 at four academic centers in Switzerland, Italy, and Israel. Eligible patients had FIGO IV disease at diagnosis and complete clinical data. Patients were grouped according to metastatic pattern: SMJN or other distant sites. Survival outcomes were analyzed using Kaplan–Meier curves and Cox proportional hazards models. **Results:** Eighty-seven patients met inclusion criteria, including 23 (26.4%) with SMJN and 64 (73.6%) with other FIGO IV metastases. Median age and histologic subtype distribution were similar between groups. Complete cytoreduction was achieved in 65.2% of SMJN patients. Median OS was not reached in the SMJN group versus 47.2 months in controls (HR 0.44; 95% CI 0.17–1.12; *p* = 0.084). Median RFS was 42.6 vs. 23.2 months, respectively (HR 0.68; 95% CI 0.35–1.32; *p* = 0.25). **Conclusions:** Patients presenting with SMJN may represent a potentially resectable manifestation within the heterogeneous spectrum of FIGO stage IV ovarian cancer, with a non-significant trend toward more favorable survival outcomes compared with other stage IV presentations.

## Simple Summary

A Sister Mary Joseph nodule is a visible tumor at the belly button that sometimes occurs in women with advanced ovarian cancer. Although it is classified as distant metastatic disease, its location suggests it may spread differently from other stage IV metastases, such as those in the liver or lungs. We conducted a multicenter retrospective study describing clinical outcomes and exploring survival patterns in patients with this nodule versus other patterns of stage IV disease. Patients with a Sister Mary Joseph nodule showed a trend toward better survival outcomes. These findings suggest that not all stage IV metastases behave the same way and that the pattern of spread may carry important prognostic information, although further validation is required. Recognizing these differences could improve how clinicians interpret advanced disease and may guide future research on individualized treatment strategies for patients with ovarian cancer.

## 1. Introduction

Sister Mary Joseph’s nodule (SMJN) is a rare but distinctive clinical sign referring to an umbilical metastasis originating from intra-abdominal or pelvic malignancies. The term was coined by Sir Hamilton Bailey in 1949 to honor Sister Mary Joseph Dempsey, a surgical assistant to Dr. William Mayo, who observed the association between umbilical nodules and internal cancers during abdominal surgeries at St. Mary’s Hospital in Rochester, Minnesota [1,2].

While uncommon, SMJN has been described in approximately 1–3% of all intra-abdominal and pelvic malignancies [3,4]. Among patients with SMJN, the distribution of primary tumors differs by sex: in men, gastrointestinal cancers—particularly of the stomach, colon, and pancreas—are most common, whereas in women, gynecologic malignancies, especially ovarian carcinoma, predominate [5,6,7]. In ovarian cancer, SMJN may occasionally present as the first and only sign of disease and is commonly misdiagnosed as an umbilical hernia, thus delaying early recognition and staging by gynecological oncologists [8,9].

Several mechanisms have been proposed to explain the spread of tumor cells to the umbilicus. These include direct extension from peritoneal surfaces, lymphatic dissemination, hematogenous spread, or migration via embryologic remnants such as the urachus or omphalomesenteric duct [10,11,12]. Iatrogenic dissemination through laparoscopic port sites has also been reported [13].

Clinically, SMJN typically presents as a firm, painless, and sometimes erythematous or violaceous nodule in the umbilical region. Although it can be mistaken for benign dermatologic conditions or hernias, its presence should raise suspicion for advanced malignancy and additional imaging should be initiated [14,15]. Accurate diagnosis requires histopathological confirmation, often aided by immunohistochemistry to identify the primary tumor site [16,17].

Historically, the appearance of SMJN has been considered an ominous prognostic sign, typically associated with widespread disease and poor survival outcomes [18,19]. Accordingly, current FIGO guidelines consider the presence of umbilical metastasis as a criterion for stage IVb disease, reflecting its classification as distant and cutaneous metastasis [20,21,22]. However, this classification raises an important clinical question: does an umbilical metastasis—often visually identifiable and potentially amenable to surgical resection—carry the same prognostic implications as visceral metastases such as those involving the lung or liver? Case reports across various tumor types suggest that the prognostic significance of SMJN may be more nuanced, raising the possibility that not all manifestations of stage IV disease behave similarly [23,24,25]. Similar favorable outcomes have also been reported, albeit rarely, in non-gynecologic malignancies such as gastric and breast cancers, particularly in patients selected for intensive therapy [26,27]. These observations challenge the traditional perception of SMJN as a uniformly terminal event and raise the possibility that, at least in some patients, SMJN may represent a distinct clinical subset of disease with less aggressive behavior or more favorable response to treatment.

Despite these insights, data on SMJN in ovarian cancer remain limited. Importantly, FIGO stage IV ovarian cancer encompasses a heterogeneous group of metastatic patterns with differing biological behavior, resectability, and treatment implications. Patients presenting with SMJN may represent a clinically and prognostically distinct subgroup among those with stage IV ovarian cancer.

This hypothesis aligns with a broader effort in gynecologic oncology to refine prognostication and treatment decisions beyond traditional anatomical staging. The ongoing TRUST trial (ENGOT-ov33/AGO-OVAR OP.7) investigates whether patients with FIGO stage IIIB–IVB ovarian cancer benefit more from primary cytoreductive surgery or from neoadjuvant chemotherapy [16,17]. Preliminary analyses suggest that, under optimal surgical conditions, patients with FIGO IIIb disease derive greater benefit from initial debulking, whereas those with FIGO IV disease may be better treated with neoadjuvant chemotherapy. Although Sister Mary Joseph nodules are formally classified as FIGO IVb, they represent a resectable manifestation rather than a distant, unresectable metastasis. This raises the possibility that SMJN represents a distinct clinical presentation within FIGO IV disease that may warrant further investigation.

In this context, the present study aims to describe clinical outcomes in patients presenting with Sister Mary Joseph nodules as a potentially resectable manifestation of FIGO stage IV ovarian cancer and to explore differences relative to other stage IV presentations in a hypothesis-generating framework.

## 2. Materials and Methods

We conducted a retrospective multicenter cohort study at four academic medical centers: University Hospital Basel (USB) and Geneva University Hospitals (HUG) in Switzerland, Fondazione Policlinico Universitario Agostino Gemelli (IRCCS) in Rome, Italy, and Edith Wolfson Medical Center in Holon, Israel.

The study was approved by the local institutional ethics committees at each participating center in accordance with national regulations and the Declaration of Helsinki.

We included patients diagnosed with epithelial ovarian, fallopian tube, or primary peritoneal carcinoma between 1 January 2010, and 31 December 2023. Eligible patients presented with FIGO stage IV disease at diagnosis and had complete clinicopathological and follow-up data available.

Patients were divided into two groups based on the site of further distant metastasis at initial presentation. The first group included patients who presented with only a Sister Mary Joseph nodule (SMJN), confirmed either clinically or radiologically. The second group consisted of patients with other forms of FIGO stage IVb disease, such as metastases to other areas of the skin, the pleura, liver, or lungs.

Data were extracted from electronic medical records and anonymized before analysis. Variables collected included age at diagnosis, histology and FIGO stage, treatment details (surgery, chemotherapy), recurrence and survival data and sites of metastatic disease at presentation.

We analyzed overall survival (OS), defined as the time from diagnosis to death from any cause or last follow-up, and progression-free/recurrence-free survival (PFS/RFS), defined as the time from completion of first-line treatment to disease recurrence or death.

Descriptive statistics are expressed as median [range] or as number (percentage), as appropriate.

Continuous variables were compared between study groups using the Mann–Whitney U test. Categorical variables were analyzed using the chi-squared test, or Fisher’s exact test when expected cell counts were less than 5.

Survival outcomes were evaluated descriptively using Kaplan–Meier estimates, with log-rank tests used to explore differences between groups. Hazard ratios (HR) and 95% confidence intervals were calculated using the Cox proportional hazards model. Statistical analyses were performed using R software (version 4.4.3; R Foundation for Statistical Computing, Vienna, Austria). A *p*-value < 0.05 was considered statistically significant.

## 3. Results

A total of 87 patients with stage IV epithelial ovarian, fallopian tube, or primary peritoneal cancer were included in the final analysis after excluding two cases with missing key clinical data. Among them, 23 patients (26.4%) presented with a Sister Mary Joseph nodule (SMJN) at diagnosis, while 64 patients (73.6%) had other forms of distant metastatic disease and served as the control group.

All patients in the control group were treated at University Hospital Basel. The SMJN group included nine patients from University Hospital Basel, nine patients from Fondazione Policlinico Universitario Agostino Gemelli IRCCS in Rome, three patients from Geneva University Hospitals, and two patients from Edith Wolfson Medical Center in Israel.

Baseline characteristics, including age at diagnosis and histologic subtype, were similar between groups (Table 1). The median age was 59.0 years in the SMJN group and 65.0 years in the non-SMJN group (*p* = 0.707). Histologic subtype distribution was similar between groups. In the SMJN group, 95.7% of tumors were serous, compared to 87.5% in the control group. The differences in histologic distribution were not statistically significant.

Data on completeness of cytoreduction were available for 22 of 23 SMJN patients. Complete gross resection (R0) was achieved in 15 patients (65.2%), while 7 patients (30.4%) had residual disease. These proportions are within the range of R0 rates reported for stage IV patients treated in high-volume centers.

The median follow-up duration was 24.3 months in the SMJN group and 30.0 months in the control group (*p* = 0.459), with no statistically significant difference between the groups.

**Figure 1:**
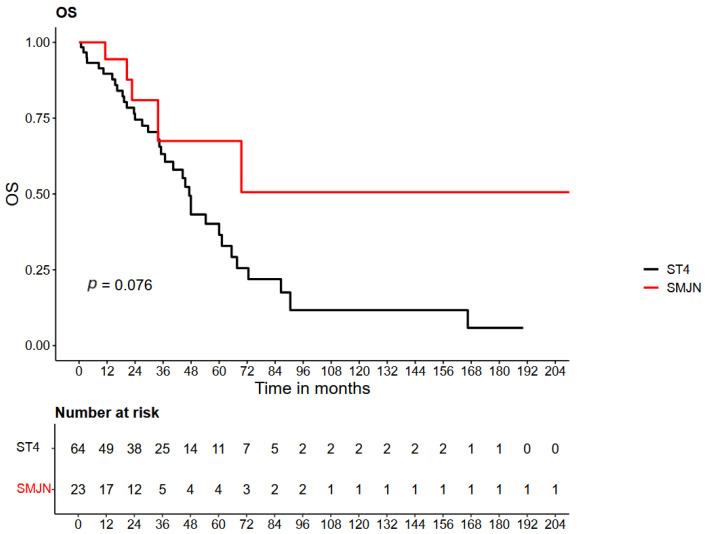
Kaplan–Meier curve of overall survival (OS) in patients with FIGO stage IV epithelial ovarian, fallopian tube, or primary peritoneal cancer, showing patients with Sister Mary Joseph nodule (SMJN) and those with other distant metastases.

In the control group, the median overall survival (OS) was 47.2 months, whereas in the SMJN group, the median OS was not reached during the follow-up period. Cox regression analysis yielded a hazard ratio (HR) of 0.436 (95% CI: 0.17–1.12; *p* = 0.084). The survival curves of both groups are shown in Figure 1.

**Figure 2:**
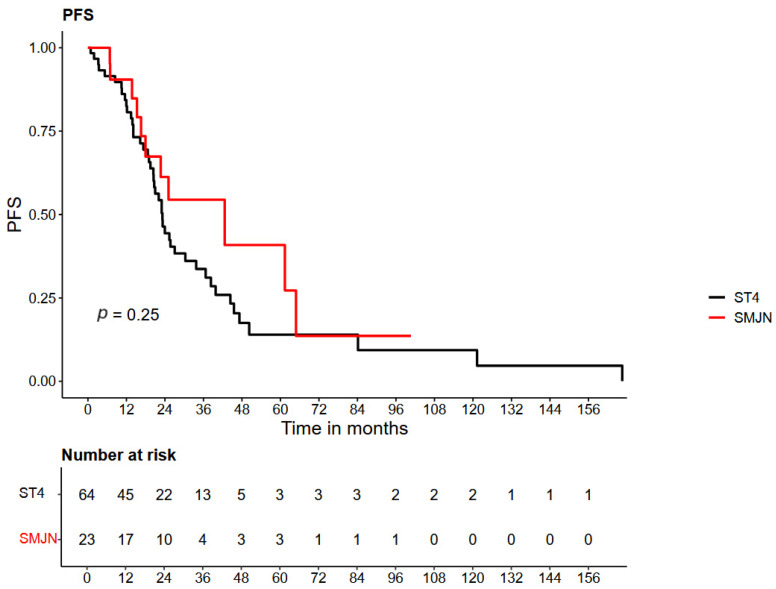
Kaplan–Meier curve of recurrence-free survival (RFS) in patients with FIGO stage IV epithelial ovarian, fallopian tube, or primary peritoneal cancer, comparing those with Sister Mary Joseph nodule (SMJN) to those with other distant metastases.

Recurrence-free survival (RFS) was also longer in the SMJN group (Figure 2). Median RFS was 42.6 months in patients with SMJN and 23.2 months in the control group. The hazard ratio for recurrence or death was 0.677 (95% CI: 0.348–1.317; *p* = 0.25), showing a non-significant trend toward improved disease control in the SMJN cohort.

## 4. Discussion

In this multicenter retrospective study, we examined outcomes in patients with various forms of FIGO stage IV epithelial ovarian, fallopian tube, or primary peritoneal carcinoma who presented either with a Sister Mary Joseph nodule (SMJN) or with other forms of distant metastasis. Although differences in RFS and OS between the two groups did not reach statistical significance, survival outcomes in patients presenting with SMJN appeared numerically more favorable in this exploratory analysis. Specifically, median OS was not reached in the SMJN group, compared to 47.2 months in the control group, and RFS was also numerically longer. These findings may indicate that SMJN represents a potentially distinct clinical phenotype within stage IV disease, warranting further investigation into its biological behavior and prognostic implications.

Since completeness of cytoreduction is a strong determinant of survival in ovarian cancer, one possible explanation for the favorable outcomes in SMJN patients could be higher R0 rates [28]. However, in our cohort, complete cytoreduction was achieved in 65.2% of SMJN patients, which is within the 60–75% range reported for stage IV patients treated in high-volume centers [18]. However, individual-level data on completeness of cytoreduction were not consistently available for the control group, precluding a direct comparison of R0 rates between cohorts. As such, differences in surgical outcomes and institutional practices cannot be excluded as contributors to the observed survival trends. Importantly, SMJN represents a readily identifiable and potentially resectable manifestation of extra-abdominal disease; however, whether any associated prognostic differences reflect underlying biological behavior or treatment-related factors cannot be determined from the present data.

The trend toward improved OS observed in patients with SMJN contrasts with the widely held perception that umbilical metastasis at presentation signifies a poor prognosis in ovarian cancer. Several case series have reported modest outcomes in SMJN, often reflecting limited treatment access or advanced disease burden at diagnosis. For example, a Tanzanian study reported a median survival of just 28 weeks among patients with SMJN, although the majority received only palliative care [19]. In contrast, other reports have documented prolonged survival, including patients with ovarian cancer who remained alive beyond 24 months following diagnosis of SMJN and received multimodal treatment [20]. One such case involved a patient with high-grade serous carcinoma and a BRCA1 mutation who responded well to platinum-based chemotherapy followed by maintenance olaparib, achieving durable disease control with no evidence of recurrence at two years [21]. These disparate findings underscore the heterogeneity of SMJN presentations and suggest that prognosis may depend more on tumor biology and treatment context than on the presence of umbilical involvement per se. Notably, in our cohort, SMJN patients had survival outcomes that appear more favorable than typically reported for FIGO stage IV ovarian cancer. In contemporary population-based cohorts, median overall survival for unselected FIGO stage IV ovarian cancer is commonly reported in the range of approximately 15 to 29 months [29,30]. However, more selected contemporary cohorts undergoing cytoreductive surgery in high-volume tertiary centers have demonstrated substantially longer outcomes, with median overall survival ranging from approximately 36 to 76 months depending on treatment strategy and completeness of resection [31], and exceeding 50 months in surgically treated patients receiving multimodal therapy in selected populations [32]. In this context, the control group outcomes observed in our study likely represent a well-treated tertiary-center population. This raises the possibility that SMJN reflects a more indolent or peritoneally confined form of metastatic disease, rather than true systemic dissemination, which could contribute to improved outcomes in appropriately treated patients.

Of note, the clinical significance of SMJN appears to vary depending on the primary malignancy. In gastrointestinal cancers such as pancreatic and cholangiocarcinoma, SMJN frequently reflects aggressive, disseminated disease and is associated with a dismal prognosis. For instance, survival in patients with SMJN from hilar cholangiocarcinoma or pancreatic cancer has generally ranged from weeks to a few months, often in palliative contexts [23,24,25]. Similarly, in endometrial cancer, although rare, SMJN typically signifies FIGO stage IV disease and is associated with metastatic spread through lymphatic or embryonic remnants, though some patients may benefit from aggressive treatment [9]. In contrast, the relatively prolonged survival observed in our ovarian cancer cohort suggests that the presence of SMJN in this context may not carry the same uniformly poor prognosis.

The trend toward improved recurrence-free survival (RFS) in SMJN patients also warrants further exploration. In our study, the median RFS was numerically longer in the SMJN group (42.6 months) compared to the control group (23.2 months). Although this difference did not achieve statistical significance, it suggests that SMJN patients may experience a less aggressive form of disease or may be less prone to widespread hematogenous or lymphatic metastasis. Previous case reports have described SMJN as being associated with peritoneal-dominant disease, with a predominantly localized spread in many instances [11,21]. This pattern of spread is distinct from the more aggressive systemic dissemination commonly seen in other stage IV ovarian cancer patients. Studies have shown that patients with isolated peritoneal metastases tend to have better responses to chemotherapy, possibly contributing to improved RFS in the SMJN group [20]. Moreover, the absence of distant visceral metastasis in SMJN cases, such as liver or lung involvement, further supports the notion that SMJN may represent a more localized metastatic disease that is amenable to cytoreductive surgery and chemotherapy.

Several anatomic and biological explanations have been proposed for the development of SMJN, which may help explain its variable prognostic implications [33]. The umbilicus represents a convergence point for blood and lymphatic flow and contains multiple embryologic remnants, rendering it anatomically contiguous with the peritoneum and connected to visceral and venous structures [34,35]. This anatomy makes it susceptible to metastatic spread through multiple potential routes, including lymphatic and hematogenous channels, direct peritoneal extension, and anatomical continuities related to embryologic remnants (e.g., urachus, ligamentum teres) [34,35]. These overlapping pathways may help explain why SMJN can arise in both disseminated and more localized disease contexts. In ovarian cancer, SMJN may in some cases arise through local peritoneal dissemination—such as spread from adjacent omental deposits to the umbilicus—rather than exclusively via systemic hematogenous or lymphatic metastasis [35]. This mechanism is consistent with our observation of relatively favorable survival outcomes. Taken together, these anatomical and biological features highlight the need to interpret SMJN in its full clinical and anatomical context.

Our findings are particularly important with the emerging data from the TRUST trial (ENGOT-ov33/AGO-OVAR OP7), a large international phase III study comparing upfront radical cytoreductive surgery followed by chemotherapy to neoadjuvant chemotherapy followed by interval debulking surgery and subsequent chemotherapy in patients with FIGO stage IIIB–IVB epithelial ovarian cancer. Preliminary results from the TRUST trial (ENGOT-ov33/AGO-OVAR OP7), presented at the 2025 ASCO Annual Meeting (Mahner S., et al. *J Clin Oncol* 43, 2025 (suppl; abstr LBA5500), suggest that patients who undergo primary surgery—when complete resection is achieved—may experience improved PFS (22.1 vs. 19.7 months; HR 0.80; *p* = 0.018) and a favorable trend in OS (54.3 vs. 48.3 months) compared to those receiving the NACT-first sequence [17]. The TRUST trial results suggest that treatment sequencing decisions in advanced ovarian cancer may be influenced not only by stage classification but also by the feasibility of achieving complete cytoreduction. However, Sister Mary Joseph nodules, although formally classified as FIGO IVb, represent a distinct and readily resectable manifestation. As such, SMJN may share biological and prognostic features with peritoneal-dominant dissemination patterns more typically associated with stage IIIB disease, rather than with visceral metastatic spread seen in other stage IV presentations. This raises the possibility that selected patients presenting with SMJN—despite formal classification as FIGO stage IVb—may benefit from aggressive upfront surgical approaches similar to those considered for patients with peritoneal-dominant disease, particularly when optimal cytoreduction is deemed feasible. Our findings are consistent with the possibility that certain patients with stage IV disease—potentially including those with SMJN—can benefit from aggressive surgical approaches when optimal cytoreduction is feasible.

Rather than representing a uniquely distinct biological entity, SMJN may be considered within a broader subgroup of FIGO stage IV ovarian cancer characterized by anatomically resectable metastatic patterns. Prior analyses of stage IV disease have shown that completeness of cytoreduction remains the dominant prognostic factor irrespective of the specific site of extra-abdominal involvement [36,37]. In this context, SMJN may represent one manifestation within a surgically controllable subset of stage IV disease, where prognosis is influenced more by resectability than by anatomical classification alone.

This study has several limitations related to its retrospective design and patient selection. First, its retrospective nature inherently carries risks of bias, including selection bias and incomplete data capture. Second, due to the rarity of SMJN at presentation, the sample size of the SMJN group was small, limiting statistical power to detect significant differences in survival outcomes. Third, all control patients were recruited from a single center, whereas SMJN cases were drawn from four institutions, potentially introducing center-related variability in diagnostic workup, treatment decisions, and follow-up protocols. Additionally, the SMJN group had a significantly later median date of diagnosis, raising the possibility of treatment-era bias. Improvements in systemic therapies over time, including the introduction of PARP inhibitors and antiangiogenic agents as maintenance strategies, may have contributed to improved outcomes in more recently treated patients. As our analysis did not adjust for year of diagnosis or treatment era, this potential confounding effect cannot be excluded. Finally, heterogeneity in treatment regimens across institutions, including variations in the use of neoadjuvant chemotherapy, surgical aggressiveness, and maintenance therapy, may have influenced patient outcomes.

Despite these limitations, the study has several notable strengths. To our knowledge, this is the first multicenter study to compare survival outcomes in ovarian cancer patients presenting with SMJN versus other forms of FIGO stage IV disease. Moreover, it represents one of the largest comparative cohorts reported to date evaluating SMJN in ovarian cancer against other stage IV metastatic patterns. The inclusion of patients from four geographically and institutionally diverse centers enhances the generalizability of the findings. Moreover, all patients were confirmed to have stage IV disease at presentation, ensuring a consistent baseline for comparison. The use of standardized definitions for recurrence and survival endpoints, along with centralized statistical analysis, further strengthens the reliability of the results. Finally, the inclusion of a relatively large number of SMJN cases, a rare clinical entity, provides a valuable contribution to a field currently dominated by single-case reports and small series.

## 5. Conclusions

Our findings suggest that SMJN may represent a potentially resectable manifestation within the heterogeneous spectrum of FIGO stage IV ovarian cancer. Although differences in OS and RFS did not reach statistical significance, the consistent trend observed in our cohort is in line with the hypothesis that SMJN may represent a clinically distinct metastatic phenotype—potentially more consistent with transcoelomic dissemination than with widespread hematogenous or lymphatic metastasis. If validated in larger future studies, these findings could have practical implications for prognosis, patient counseling, and therapeutic decision-making. For instance, patterns of metastatic spread may provide additional clinical context alongside FIGO staging when discussing expected outcomes and treatment strategies. We therefore recommend prospective multicenter studies and collaborative registries to validate these findings and to clarify whether SMJN represents a clinically meaningful subgroup within advanced ovarian cancer.

## Figures and Tables

**Table 1:** Patient clinical and pathological characteristics.

Variable	All (*n* = 87)	SMJN (*n* = 23)	Control (*n* = 64)	*p*-Value
Age at diagnosis	64.0 [22.0; 93.0]	59.0 [25.0; 92.0]	65.0 [22.0; 93.0]	0.707
Histology				0.604
Clear Cell	1 (1.15%)	0 (0.00%)	1 (1.56%)	
Endometrioid	2 (2.30%)	1 (4.35%)	1 (1.56%)	
Serous	78 (89.7%)	22 (95.7%)	56 (87.5%)	
Undifferentiated	1 (1.15%)	0 (0.00%)	1 (1.56%)	
Unknown	5 (5.75%)	0 (0.00%)	5 (7.81%)	

SMJN = Sister Mary Joseph’s Nodule.

## Data Availability

The data presented in this study are available on request from the corresponding author. The data are not publicly available due to privacy and ethical restrictions related to the use of health-related personal data, as approved by the Ethics Committee of Northwest and Central Switzerland (EKNZ, Project ID 2023-02196).
